# The impact of unhealthy food sponsorship vs. pro-health sponsorship models on young adults’ food preferences: a randomised controlled trial

**DOI:** 10.1186/s12889-018-6298-4

**Published:** 2018-12-20

**Authors:** Helen Dixon, Maree Scully, Melanie Wakefield, Bridget Kelly, Simone Pettigrew, Kathy Chapman, Jeff Niederdeppe

**Affiliations:** 1 Centre for Behavioural Research in Cancer, Cancer Council Victoria, Melbourne, Victoria Australia; 2 Early Start, School of Health and Society, University of Wollongong, Wollongong, New South Wales Australia; 3 School of Psychology, Curtin University, Bentley, Western Australia; 4 School of Life and Environment Sciences, Faculty of Science, University of Sydney, Sydney, New South Wales Australia; 5 School of Medicine and Public Health, University of Newcastle, Callaghan, New South Wales Australia; 6 Department of Communication, Cornell University, Ithaca, New York USA

**Keywords:** Sport sponsorship, Food marketing, Public health, Young adults, Nutrition, Obesity prevention

## Abstract

**Background:**

Unhealthy foods are promoted heavily, through food company sponsorship of elite sport, resulting in extensive exposure among young adults who are avid sport spectators. This study explores the effects of sponsorship of an elite sporting event by: (A) non-food brands (control), (B) unhealthy food brands, (C) healthier food brands, or (D) an obesity prevention public health campaign on young adults’ brand awareness, attitudes, image perceptions, event-sponsor fit perceptions, and preference for food sponsors’ products.

**Methods:**

A between-subjects web-based experiment was conducted, consisting of four sponsorship conditions (A through D) featuring three product categories within each condition. Australian adults (*N* = 1132) aged 18–24 years were recruited via a national online panel. Participants viewed promotional videos and news stories about an upcoming international, multi-sport event (with sponsor content edited to reflect each condition), completed a distractor task, and then answered questions assessing the response variables. Regression analyses were conducted to test for differences by sponsorship condition on the respective outcome measures.

**Results:**

Compared to the control condition, unhealthy food sponsorship promoted higher awareness of, and more favourable attitudes towards, unhealthy food sponsor brands. Unhealthy food sponsorship also led to greater perceived event-sponsor fit and transfer of perceptions of the sporting event to the unhealthy food sponsor brands, relative to the control group. Exposure to sponsorship for healthier foods produced similar sponsorship effects for healthier food sponsor brands, as well as prompting a significant increase in the proportion of young adults showing a preference for these products. Obesity prevention campaign sponsorship promoted higher campaign awareness and perceived event-sponsor fit, but did not impact food attitudes or preference for unhealthy versus healthier foods.

**Conclusion:**

Findings suggest that restricting elite sport sponsorship to healthier food brands that meet set nutritional criteria could help promote healthier eating among young adults. Sporting organisations should be encouraged to seek sponsorship from companies who produce healthier food brands and government-funded social marketing campaigns.

**Clinical trial registration:**

Australian New Zealand Clinical Trials Registry (ANZCTR) registration number ACTRN12618000368235. Retrospectively registered 12 March 2018.

## Background

Research has implicated commercial marketing of energy-dense nutrient-poor foods and beverages (collectively called ‘unhealthy food’) in contributing to population levels of overweight and obesity and poor diet [1]. Considerable public health research and policy debate has centred on unhealthy food marketing to children (especially product advertising on television), with a substantial body of evidence indicating food promotions have a direct effect on children’s nutrition knowledge, preferences, purchase behaviour, consumption patterns and diet-related health [1, 2]. Although young people are susceptible to influence by unhealthy food marketing [3], comparably little public health attention has been directed to assessing the impacts of prominent forms of unhealthy food marketing targeted at adolescents and young adults. Many Australian young adults’ diets are sub-optimal [4], placing them at increased risk of diet-related chronic disease in later life [1]. The lifestyle changes young adults face during the transition from adolescence to adulthood can make them vulnerable to declines in diet quality and weight gain [5, 6], rendering it a particularly important life stage for establishing and intervening on long-term health behaviours. To help address this gap, the current study assessed young adults’ responses to elite sport sponsorship by unhealthy food brands. It also explored the efficacy of using alternative, pro-health sport sponsorship models to improve young adults’ dietary choices; in recognition that elite sport sponsorship is a potentially modifiable environmental factor.

### Sponsorship exposure

Elite sport sponsorship achieves high reach and powerful engagement with young adults through live spectatorship and mass media. Around half of all Australians aged 18–24 years attend at least one sporting event a year [7]. Further, around half of the Australian population watches sport on commercial free-to-air television every week [8]. Food and beverage companies exploit elite sports’ marketing potential, with prevalent sponsorship of various Australian sports through their governing bodies by companies promoting unhealthy food, alcohol and gambling products. This has led to calls for regulatory guidelines to be established to limit such sponsorship [9]. At the same time, opportunity exists for public health organisations to capitalise on elite sport sponsorship for reaching young adults with well-aligned healthier eating and body weight messages.

Elite sport sponsorship involves multiple complementary marketing strategies such as advertising at sporting events (e.g. perimeter and tier boards, signage around scoreboards, painted or superimposed logos on field); logos on uniforms; promotional clothing for players, coaches and fans; naming of a series, game or stadium; exclusive product category sale rights at events; commercial break advertisements in broadcasts; and product endorsements by sport stars [10]. In Australia, businesses invest an estimated AUD$774 million in sport sponsorship annually, 6% of which is from soft drink companies; this is a higher percentage than in most other countries [11]. Unhealthy food brands receive immense exposure from elite sport sponsorship. For example, during a sample of cricket broadcasts in the summer of 2014, 3372 incidences of unhealthy food promotion (including fixed advertising, dynamic advertising, commercials, integrated advertising and team sponsorship) occurred [12] – mostly for KFC, the “official fast food restaurant” of Cricket Australia and naming rights sponsor for Twenty20 cricket in Australia. Internationally, both McDonald’s and Coca-Cola have been long-standing partners of the Olympic Games and the FIFA World Cup, leading to high visibility of their brands to the billions of people worldwide who watch these sporting events [13].

### Impacts of sponsorship

Research indicates that sponsorship can increase brand awareness, modify brand image and increase intentions to purchase sponsor products [14–16]. Similarly, health messages within a comprehensive sponsorship program can successfully build awareness and behavioural intention among people attending sport and arts events [17]. Exposure to elite sport sponsorship by alcohol and tobacco brands is positively associated with consumption of sponsors’ products [15, 18].

Efforts to explain these findings typically consider the relationship between people, sponsors and events. Image transfer refers to the idea that favourable attitudes toward an event transfer over to favourable attitudes toward the sponsoring brand, providing consumers who are favourably disposed to that image a reason to purchase the sponsoring brand [19]. This image transfer can create a “health halo” by passing on positive images of sport to brands, such as health, youth, energy and peak performance [20, 21]. Sport sponsorship provides a powerful opportunity to strengthen brand attachment and brand image, as well as to enhance public perceptions of good corporate citizenship [14, 22, 23].

Sponsorship may be an especially persuasive marketing strategy because it engages the consumer differently to other forms of advertising and promotion. Sport sponsorship can bestow the benefit of an activity with which the consumer often has an intense emotional relationship onto a brand [24]. Strong engagement and affiliation with an activity can thus create emotional bonding with the brand. Personal liking and perceived status of the sponsored event are positively associated with a favourable response to sports sponsorship [25]. Studies with spectators at different sporting events have found that high levels of event identification increase image transfer perceptions [19].

Event-sponsor fit (i.e. spectators’ perception of a logical connection between event and sponsor) has also been theorised as important to whether image transfer occurs [24–26], and research consistently suggests that such congruence is a positive predictor of effective sponsorship [27, 28]. Perceptions of event-sponsor fit are positively associated with interest in the sponsor, attitude toward the sponsor and intention to use the sponsored product [25]. Some event-sponsor pairings have an obvious basis for connection (e.g. Valvoline sponsoring motor car racing) that should increase image transfer [19]. In the case of unhealthy food sponsorship of sport, however, it is unclear whether consumers see a logical event-sponsor fit (e.g. high energy food gives you energy for sport) or not (e.g. unhealthy food undermines athletic performance or has nothing to do with sport itself). Nutritious foods and health promotion messages seem more plausibly associated with sport than unhealthy foods.

### Objectives and hypotheses

Theory and research on sponsorship provide insights into how unhealthy food sport sponsorship influences consumers and elucidate potential methods for countering this process. Potential methods for reorienting sport sponsorship away from promoting unhealthy eating towards promoting healthier eating could include replacing unhealthy food sponsorship with sponsorship by non-food brands, healthier food brands or public health campaigns promoting healthier eating and body weight. The present project explored young adults’ responses to unhealthy food sponsorship, as well as various alternative pro-health sponsorship scenarios to provide insight into the extent to which sponsorship replacement strategies would be effective in promoting awareness and preference for healthier sponsor brands. Specifically, it aimed to test the effects of sponsorship of an elite sporting event by: (A) non-food brands (conceptualised as the control condition); (B) unhealthy food brands; (C) healthier food brands; or (D) an obesity prevention public health campaign on young adults’ brand awareness; attitudes; image perceptions; event-sponsor fit perceptions; and preference for food sponsors’ products.

It was hypothesised that unhealthy food sponsorship would promote higher awareness of (H1a) and more favourable attitudes towards unhealthy food sponsor brands (H1b), stronger perceptions of fit between the sporting event and unhealthy food sponsor brands (H1c), greater transfer of the sporting event’s image to unhealthy food sponsor brands (H1d) and higher preference for unhealthy food sponsor branded products (H1e) relative to non-food sponsorship. Similarly, it was hypothesised that healthier food sponsorship would promote higher awareness of (H2a) and more favourable attitudes towards healthier food sponsor brands (H2b), stronger perceptions of fit between the sporting event and healthier food sponsor brands (H2c), greater transfer of the sporting event’s image to healthier food sponsor brands (H2d) and higher preference for healthier food sponsor branded products (H2e) compared to non-food sponsorship. It was also expected that exposure to obesity prevention campaign sponsorship would promote higher awareness of the campaign (H3a) and stronger perceptions of fit between the sporting event and the campaign (H3b) than exposure to non-food sponsorship. Additional research questions tested whether obesity prevention campaign sponsorship would promote less favourable attitudes towards (RQ1a) and reduced preference for (RQ1b) unhealthy foods, and more favourable attitudes towards healthier foods (RQ1c) compared to the control condition, whether unhealthy food sponsorship would promote higher preference for unhealthy foods overall, or simply increased preference for the sponsor’s brand of unhealthy food (RQ2), and whether healthier food sponsorship would promote reduced preference for unhealthy foods overall (RQ3).

## Method

### Design and procedure

A between-subjects web-based experiment was conducted whereby young Australian adults were randomly assigned to one of the four sponsorship conditions (A to D) and then further randomised to one of three product categories within condition, using a programming script in the survey. For example, in conditions B and C the product categories tested included breakfast cereals, take-away foods, and non-alcoholic beverages, which are all commonly marketed through sport sponsorship. Participants first viewed promotional videos for the 2018 Commonwealth Games and associated fictional news stories, with content edited to reflect their assigned sponsorship condition, then completed questions assessing their perceptions of this major sporting event. Next, participants completed a logic puzzle as a distractor task before answering a series of questions assessing their brand awareness, attitudes and image perceptions, event-sponsor fit perceptions and their preference for food sponsor products. Approval to conduct the study was obtained from Cancer Council Victoria’s Institutional Research Review Committee (IER 1606).

A sample of Australian adults aged 18 to 24 years (‘young adults’) was recruited from two opt-in online panels managed by i-Link Research (http://i-link.com.au) and Survey Sampling International (https://www.surveysampling.com). Both panels offer member points for completing surveys that can be redeemed for rewards (e.g. shopping gift cards). Panellists from i-Link Research were approached via a direct email invitation for our survey, while those from SSI received a general email invitation to participate in an “active survey” and then answered a set of profiling questions to enable them to be randomly matched to a survey they were likely to be eligible to complete. Upon accessing our survey individually at a time and location of their convenience, panellists were asked two screening questions to confirm they met the age eligibility criteria and to determine their frequency of engagement with media coverage of sport. Panellists who reported not watching, reading or listening to any media coverage of sport were excluded from participating. Quotas were applied to achieve approximately even numbers of males and females within conditions. Based on previous experimental media research [29, 30], we expected our intervention to produce small effect sizes. To detect group differences of this magnitude at 80% power and α = 0.05, a sample size of *n* = 274 per condition is required [31]. Thus, we aimed to recruit *N* = 1096 young adults into our study (*n* = 274 × 4 conditions). Participants were blind to their assigned sponsorship intervention, with the introduction to the online survey simply stating, “As part of this survey, you will be asked to view two advertisements and read some short news stories before answering a series of questions”.

### Sponsorship stimuli

To maximise participants’ engagement with the intervention, a combination of audio-visual and written communication stimuli were used to portray the fictional sponsorship relationship between the upcoming 2018 Commonwealth Games and each particular sponsor brand. We chose the Commonwealth Games as it is a discrete, elite sporting event being held in Australia and, given the timing of data collection (November to December 2016), participants were unlikely to hold pre-conceived notions of the actual sponsors due to the substantial time period until the event (April 2018).

Two existing promotional videos for the 2018 Commonwealth Games were sourced online via YouTube (1 × 35 s (https://www.youtube.com/watch?v=H8hVYvPViHI) and 1 × 15 s (https://www.youtube.com/watch?v=sYsf1VGXD1o) in length) and professionally edited to include a new end-frame. This end-frame featured the fictional sponsor brand logo along with “Official Partner” text next to the Games logo, with the slogan “Come share the dream with us” underneath.

A graphic designer developed the layout and design for two mock online news pages about the Games. The first news page included an article profiling four Australian athletes the public should watch out for at the 2018 Commonwealth Games. The second news page reported on the announcement of the brand as a major sponsor of the Games. Two of the researchers (MS and HD) wrote the text for the news articles, with content informed by public materials about the athletes and the Games, which was then edited by a communications expert for clarity and conciseness. A static banner advertisement for the Games, featuring the assigned fictional sponsor brand logo, was shown at the bottom of both news pages. The second news page also included a prominently placed image of the Games logo next to a real-life advertisement for the assigned fictional sponsor brand (sourced online) to highlight the new partnership.

Each intervention component appeared on a separate screen within the online survey. The average duration of the entire intervention was 1 min and 45 s.

The food brands tested in the sponsorship simulation were chosen because they are prominent in the Australian marketplace within a particular product category. While some of these brands produce a mixture of unhealthy and healthier products, their use in a particular sponsorship condition was based on the overall nutritional profile of the majority of foods they sell and promote under that brand, and that they produce some products that exemplify either unhealthy or healthier options. The respective food sponsor brands tested in our sponsorship simulation were: a breakfast cereal brand that produces a number of prominent unhealthy (esp. high sugar) cereal products; a healthier breakfast cereal brand that produces a number of healthier cereal products that are high in fibre and low in sugar, salt and fat; an unhealthy take-away food brand that sells burgers, fries, sugary drinks and desserts; a healthier take-away food brand that sells sandwiches and salads of varying levels of healthfulness, but healthier options are available (e.g. wholegrain roll, fresh salad, lean meat); an unhealthy beverage brand of sugary lemon-lime flavoured soda; a healthier beverage brand of bottled spring water containing no sugar.

In the obesity prevention campaign sponsorship condition, we tested a past national healthier eating campaign promoting the recommended daily intake of fruit and vegetables) [32], a healthier weight and lifestyle campaign that has run in some Australian states [33], and a national awareness-raising campaign regarding sugar-sweetened beverages [34]. The control condition included a well-known bank, airline and telecommunications brand. (Note that our experimental simulation featured fictional sponsors of the 2018 Commonwealth Games for research purposes; participants were debriefed on this at the conclusion of the study. For a list of actual 2018 Commonwealth Games sponsors see https://www.gc2018.com/sponsors.)

### Measures

#### Brand awareness

Participants were asked to list up to three brands that came to mind when they thought about either breakfast cereals, take-away foods or non-alcoholic drinks (depending on their assigned product category). In addition, all participants were asked, “When you think of public health campaigns aimed at encouraging Australian adults to eat a healthier diet or be more active, which ones come to mind?” Participants could nominate up to three public health campaigns, and for both questions there was an option of “none”. Open-ended responses were coded to create binary variables indicating whether or not participants were aware of the: unhealthy food sponsor brand; healthier food sponsor brand; and obesity prevention campaign sponsor brand, for their assigned product category.

#### Brand attitudes

Participants were asked about their attitude toward each food sponsor brand (the three unhealthy and three healthier food sponsor brands; six in total) using three 7-point semantic differential scales anchored by negative/positive, unfavourable/favourable and bad/good [35]. These items formed reliable scales for each food sponsor brand (Cronbach’s α ranged from .92 to .94). Separate brand attitude variables were created to reflect participants’ average rating of the particular unhealthy food sponsor brand and healthier food sponsor brand, for their assigned product category. To compute overall measures of attitudes towards unhealthy food brands and healthier food brands in general, we averaged ratings of the three unhealthy food sponsor brands and three healthier food sponsor brands, respectively.

#### Event-sponsor fit

Using a scale developed by Speed and Thompson [25], participants were asked to indicate the extent to which they agreed (1 = strongly disagree to 7 = strongly agree) with five statements assessing the level of fit between the sponsored sporting event and the: unhealthy food sponsor brand; healthier food sponsor brand; and obesity prevention campaign brand, for their assigned product category. The five statements included: “There is a logical connection between the Commonwealth Games and [sponsor brand]”; “The image of the Commonwealth Games and the image of [sponsor brand] are similar”; “[Sponsor brand] and the Commonwealth Games fit together well”; [Sponsor brand] and the Commonwealth Games stand for similar things]; and “It makes sense to me that [sponsor brand] are sponsoring the Commonwealth Games” (Cronbach’s α ranged from .94 to .97).

#### Event/brand image similarity

Following exposure to the intervention, participants were asked to rate how well (1 = not at all to 7 = very well) each of the following 10 adjectives describes the 2018 Commonwealth Games: exciting, active, healthier, elite, inspiring, fun, patriotic, competitive, energetic and tough. We identified the final set of 10 adjectives based on results from pre-testing of 25 adjectives (generated by the research team) with a convenience sample of 13 young adults to determine their usefulness in describing the 2018 Commonwealth Games. In a later section of the questionnaire, participants were asked to rate how well the same 10 adjectives describe the unhealthy and healthier food sponsor brands for their assigned product category. As per Gwinner and Eaton [36], we calculated measures of event/brand image similarity by summing the absolute differences between participants’ ratings of the Commonwealth Games and the (a) unhealthy food sponsor brand and (b) healthier food sponsor brand, on each of the 10 adjectives. We then reverse coded the summed absolute difference score for each event-brand pair such that higher numbers in the index indicated greater similarity.

#### Brand preferences

For each product category, participants were shown images of two unhealthy and two healthier products (with their corresponding brand logos), including one product from the respective food sponsors featured in conditions B and C, and asked to choose which one they would most prefer to buy (see Table 1). Insofar as possible, the non-sponsor products were comparable to the sponsor products with equivalent overall nutritional profiles as judged by their health star rating [37] within the healthier and unhealthy pairs. The Australian Health Star Rating system is a government-endorsed system used to summarise the overall nutritional profile of a packaged food from ½ a star to 5 stars, with more stars indicating a healthier choice [38]. A product’s health star rating is calculated using the official “Health Star Rating Calculator” which takes into account total energy (kilojoules), the quantity of ingredients linked to increased chronic disease risk (e.g. saturated fat, sodium, sugar) as well as the amount of healthier ingredients (e.g. fibre, protein, fruit, vegetable, nut and legume content). Table 1 lists the Health Star Rating for each of the food products featured in the brand preference task to summarise their overall nutritional profile to readers. However, study participants were only shown images of these products (with no Health Star Rating) when undertaking the brand preference task. We created separate binary variables to indicate whether or not participants selected the unhealthy food sponsor branded product or healthier food sponsor branded product, for their assigned product category, and a count variable to indicate the number of unhealthy foods each participant selected (range: 0–3).

**Table 1:** Sponsor and non-sponsor branded food products used in brand preferences task, with health star rating^a,b^

	Unhealthy product		Healthier product	
Product category	Sponsor brand	Non-sponsor brand	Sponsor brand	Non-sponsor brand
Breakfast cereal	Sugary cereal★★	Sugary cereal★★	Healthier cereal★★★★	Healthier cereal★★★★
Take-away food	Chicken burger★★★	Chicken burger★★	Chicken & salad roll★★★★	Chicken & salad roll★★★★
Non-alcoholic beverage	Sugary lemonade★	Sugary lemonade★	Mineral water★★★★★	Mineral water★★★★★

^a^Health Star Ratings were used by the research team to help identify unhealthy vs. healthier products within a product category; they were not shown to study participants

^b^This table describes brands and products in generic terms. Actual brand names and product images were displayed to participants undertaking the brand preference task

#### Baseline characteristics

Data on participants’ sex, age, educational status, residential postal code and parental status were collected. Socio-economic position (SEP) was estimated according to the Australian Bureau of Statistics’ Index of Relative Socio-Economic Disadvantage, based on participants’ residential postal code [39]. Participants who resided in a postcode ranked in the bottom third of the index were categorised as low SEP, those in the middle third of the index as medium SEP, and those in the upper third as high SEP. Self-reported height and weight were assessed to enable computation of participants’ body mass index [weight (kg)/height (m)^2^], since some studies have found that people who are obese may respond differently to food cues and food marketing than those with lower BMI [40–43], and we did not want this baseline characteristic to confound the experimental results. Participants were asked to report their usual frequency of consuming products from the unhealthy and healthier sponsor brands featured in their allocated product condition (i.e. breakfast cereal, take-away food, or non-alcoholic beverage). Response options included ‘every day’, ‘a few times a week’, ‘a few times a month’, ‘a few times a year’ and ‘never’.

### Statistical analysis

Data were analysed using Stata/MP V.14.2 [44]. Preliminary analyses were performed to assess whether random assignment to sponsorship condition yielded equivalent demographic groups. A combination of linear (for continuous variables), logistic (for binary variables) and Poisson (for count variables) regression analyses were used to test for differences by sponsorship condition on each of our outcome measures. All models specified the non-food sponsorship (control) condition as the reference category and included product category as a covariate. Predicted proportions and predicted means calculated from these covariate-adjusted models are reported throughout the results.

## Results

### Sample characteristics and group assignment

**Fig. 1:**
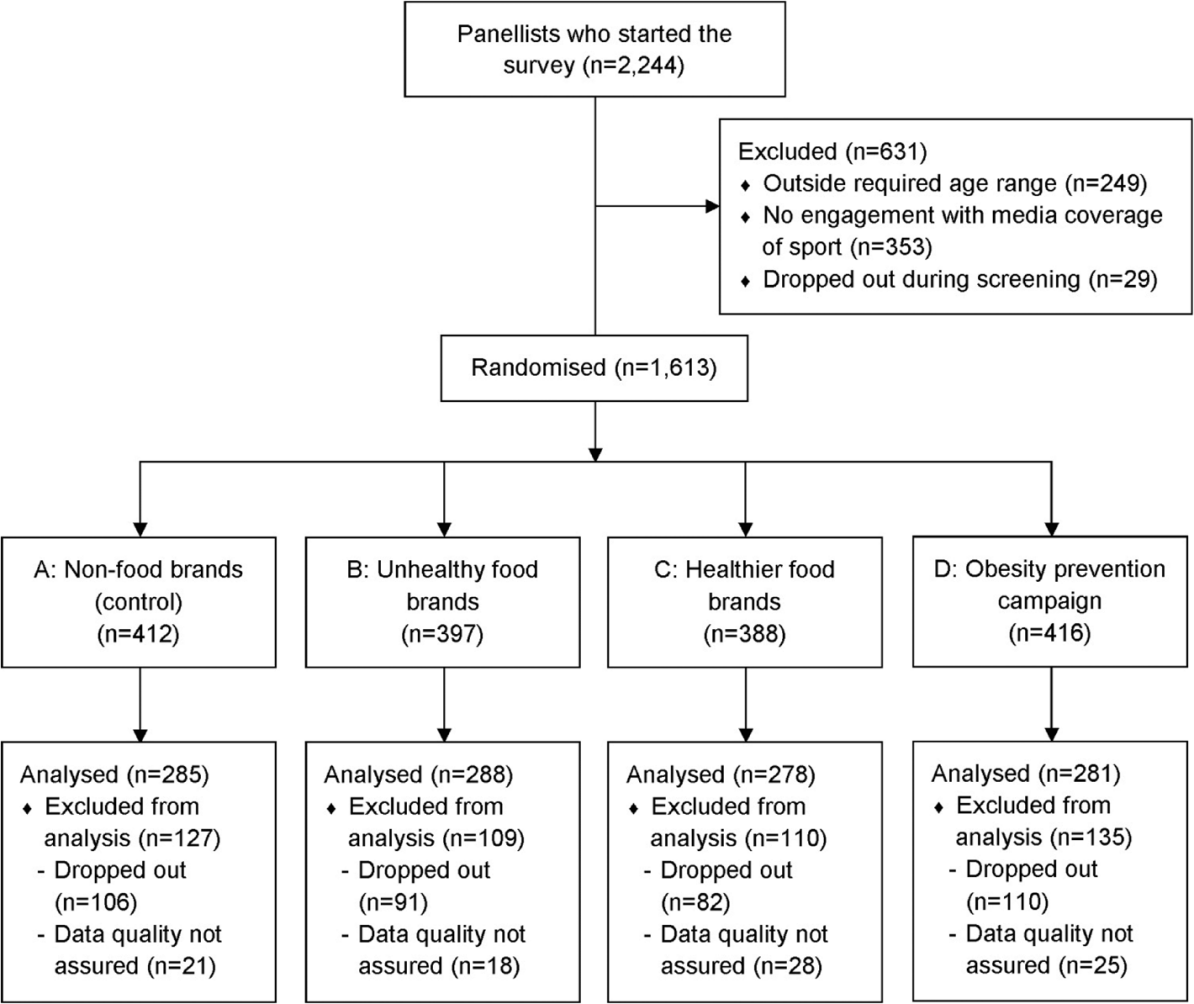
CONSORT flow diagram

A total of 2244 panellists accessed the survey between 16th November and 13th December 2016. Of these, 631 were excluded prior to randomisation due to being outside the required age range (*n* = 249), reporting they had no engagement with media coverage of sport (*n* = 353), or dropping out during screening (*n* = 29). After accounting for incomplete surveys (*n* = 389) and cases removed following standard quality control processes (*n* = 92), a final sample of 1132 eligible young adults was available for analysis (see Fig. 1 for CONSORT flow diagram).

Table 2 summarises the demographic profile of the sample. Overall, 47% of participants were male, just over half (54%) were either currently undertaking or had completed a tertiary degree, and a quarter resided in a low SEP neighbourhood [39]. The mean age of participants was 21 years. Only 13% of participants were the parent or carer of a child. One in five participants was classified as overweight while a further 10% were obese, indicating slightly lower overweight/obesity prevalence than recent national survey data for young adults [45]. Nineteen percent of participants reported never consuming the unhealthy food sponsor brand, whereas 30% indicated that they did not eat or drink the healthier food sponsor brand. The majority of participants followed media coverage of sport at least weekly (36%) or on a daily basis (33%). No significant differences in demographic characteristics were found across sponsorship conditions, indicating successful randomisation to conditions.

**Table 2:** Sample characteristics by sponsorship condition

	Total (*N* = 1132)	Sponsorship condition				Test statistic
		Non-food branding (control) (*n* = 285)	Unhealthy food branding (*n* = 288)	Healthier food branding (*n* = 278)	Obesity prevention campaign branding (*n* = 281)	
Sex						
Male	47.0%	47.7%	46.5%	46.8%	47.0%	χ^2^(3) = 0.09, *p* = 0.993
Female	53.0%	52.3%	53.5%	53.2%	53.0%	
Age						
Mean (SD)	21.0 (2.1)	20.8 (2.0)	21.0 (2.1)	21.0 (2.1)	21.0 (2.0)	F(3) = 0.75, *p* = 0.520
Educational status						
Primary/secondary only	25.6%	22.5%	27.4%	25.9%	26.7%	χ^2^(6) = 4.37, *p* = 0.627
Undertaking/completed TAFE or Trade Certificate/Diploma	20.7%	22.8%	21.2%	21.2%	17.4%	
Undertaking/completed university or other Tertiary Institute degree	53.7%	54.7%	51.4%	52.9%	55.9%	
SEP (area-based)^a^						
Low	24.6%	22.8%	25.0%	23.4%	27.4%	χ^2^(6) = 6.66, *p* = 0.354
Medium	36.7%	36.1%	33.0%	38.8%	39.1%	
High	38.6%	41.1%	42.0%	37.8%	33.5%	
Parent/carer of child (< 18 years)						
Yes	13.0%	11.2%	14.6%	14.0%	12.1%	χ^2^(3) = 1.89, *p* = 0.595
No	87.0%	88.8%	85.4%	86.0%	87.9%	
Body mass index (BMI) category^b^						
Healthier weight/underweight	69.8%	71.9%	66.7%	68.6%	72.3%	χ^2^(6) = 3.68, *p* = 0.719
Overweight	20.0%	18.2%	23.8%	19.5%	18.4%	
Obese	10.1%	9.9%	9.5%	11.9%	9.2%	
Frequency of consuming unhealthy food sponsor brand						
Every day / A few times a week	15.0%	14.7%	16.0%	16.2%	13.2%	χ^2^(9) = 3.22, *p* = 0.955
A few times a month	29.5%	29.5%	30.9%	27.3%	30.2%	
A few times a year	36.6%	36.5%	36.8%	36.7%	36.3%	
Never	18.9%	19.3%	16.3%	19.8%	20.3%	
Frequency of consuming healthier food sponsor brand						
Every day / A few times a week	15.7%	10.9%	16.7%	19.4%	16.0%	χ^2^(9) = 8.97, *p* = 0.440
A few times a month	25.2%	27.0%	24.0%	24.5%	25.3%	
A few times a year	29.4%	30.9%	28.1%	28.4%	30.2%	
Never	29.7%	31.2%	31.3%	27.7%	28.5%	
Frequency of engagement with media coverage of sport						
Daily	33.0%	32.6%	34.0%	29.5%	35.6%	χ^2^(9) = 6.75, *p* = 0.663
At least weekly	35.5%	34.4%	35.8%	34.9%	37.0%	
At least monthly	11.4%	12.6%	10.1%	14.0%	8.9%	
Less often than monthly	20.1%	20.4%	20.1%	21.6%	18.5%	

Percentages may not sum to 100% due to rounding

^a^SEP was determined according to the Australian Bureau of Statistic’s Index of Relative Socio-Economic Disadvantage ranking for Australia using participant’s residential postcode

^b^BMI information is missing for 303 participants as they did not self-report their height and/or weight

### Brand awareness

**Fig. 2:**
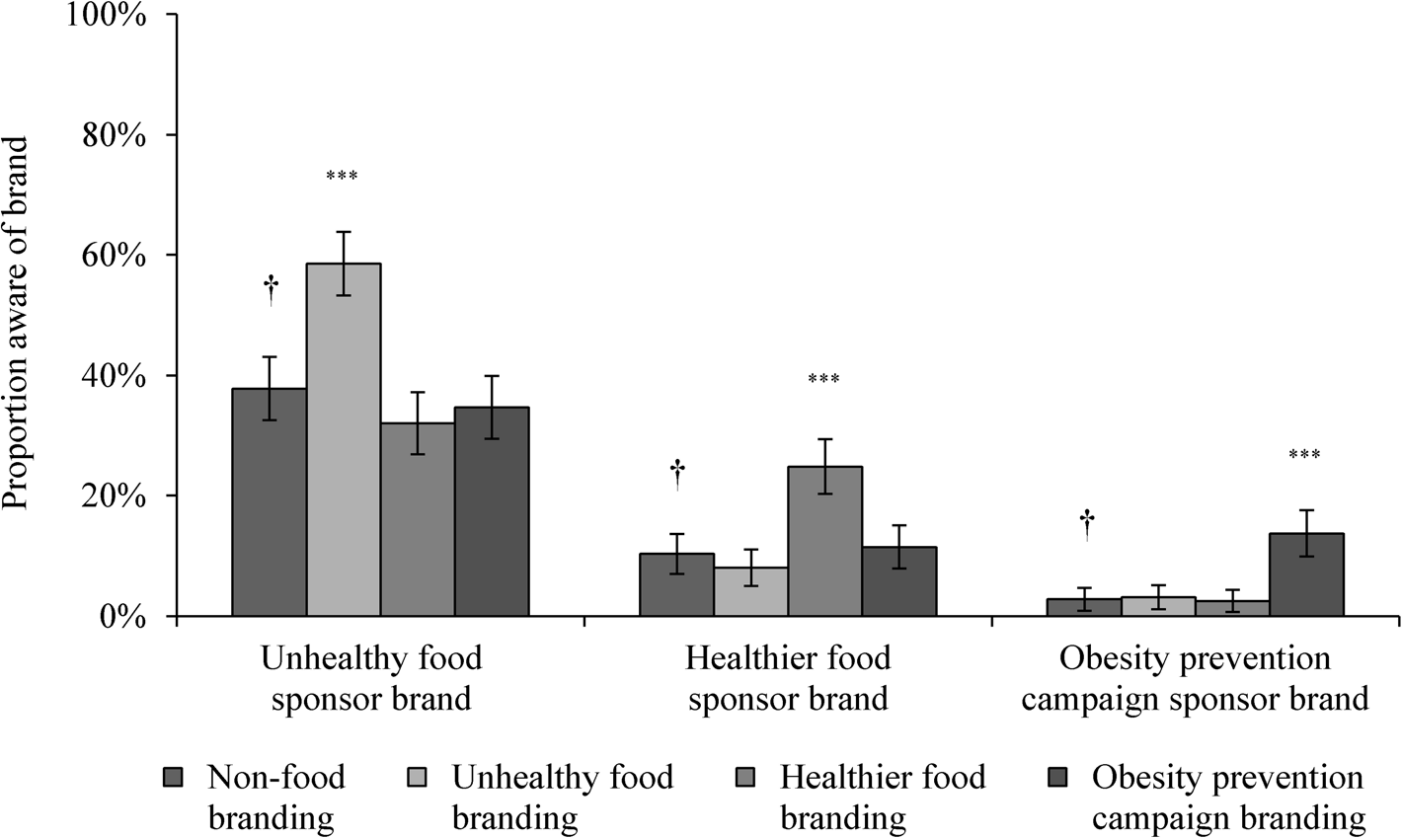
Predicted proportion of young adults with awareness of sponsor brands by sponsorship condition. *Notes*: ^†^ reference category for logistic regression analyses; ^*^ *p* < 0.05; ^**^ *p* < 0.01; ^***^ *p* < 0.001. Logistic regression analyses included product category as a covariate

Figure 2 displays the proportion of young adults in each condition who were aware of the unhealthy food sponsor brand, healthier food sponsor brand and obesity prevention campaign sponsor brand, respectively. As hypothesised (H1a), participants exposed to unhealthy food sponsorship had higher unprompted awareness of the unhealthy food sponsor brand than those exposed to non-food sponsorship (58.6% vs. 37.8%; OR = 2.63, 95% CI: 1.84–3.78, *p* < 0.001). Compared to the non-food sponsorship condition, participants in the healthier food sponsorship condition had higher unprompted awareness of the healthier food sponsor brand (supporting H2a; 24.8% vs. 10.4%; OR = 3.41, 95% CI: 2.06–5.64, *p* < 0.001), and the odds of being aware of the obesity prevention campaign sponsor brand were higher among participants exposed to this type of sponsorship (supporting H3a; 13.8% vs. 2.8%; OR = 5.99, 95% CI: 2.71–13.27, *p* < 0.001).

### Brand attitudes

Overall, participants’ attitudes towards the food sponsor brands were positive, albeit more favourable for the healthier food sponsor brand than the unhealthy food sponsor brand (M = 5.15 vs. M = 4.82; t(1131) = 6.18, *p* < 0.001). As presented in Table 3, participants held more favourable attitudes towards the unhealthy food sponsor brand following exposure to unhealthy food sponsorship compared to non-food sponsorship (supporting H1b; B = 0.41, β = 0.12, *p* < 0.001). Similarly, exposure to healthier food sponsorship was associated with more favourable attitudes towards the healthier food sponsor brand (supporting H2b; B = 0.29, β = 0.09, *p* = 0.010).

**Table 3:** Attitudinal ratings of unhealthy and healthier food sponsor brands^~^ by sponsorship condition

	Unhealthy food sponsor brand				Healthier food sponsor brand			
Sponsorship condition	Predicted mean	B (95% CI)	β	p	Predicted mean	B (95% CI)	β	p
Non-food branding	4.65	Ref			5.05	Ref		
Unhealthy food branding	**5.07**	**0.41 (0.18, 0.64)**	**0.12**	**< 0.001**	5.09	0.04 (−0.17, 0.26)	0.01	0.682
Healthier food branding	4.85	0.20 (−0.04, 0.43)	0.06	0.098	**5.34**	**0.29 (0.07, 0.50)**	**0.09**	**0.010**
Obesity prevention campaign branding	4.72	0.06 (−0.17, 0.30)	0.02	0.583	5.12	0.07 (−0.14, 0.29)	0.02	0.498

^~^Attitudes towards the obesity prevention campaign brands were not assessed. *B* unstandardised regression coefficient, *CI* confidence interval, *β* standardised regression coefficient, *Ref* reference category in linear regression model. Linear regression analyses included product category as a covariate. Boldfaced results are significant at *p* < 0.05

Additional exploratory analyses (data not shown in table) indicated there were no significant differences between the obesity prevention campaign sponsorship and non-food sponsorship conditions in terms of participants’ attitudes towards unhealthy food brands (RQ1a) and healthier food brands (RQ1c) generally (both *p* > 0.05). However, participants exposed to unhealthy food sponsorship held more favourable attitudes towards unhealthy food brands than those exposed to non-food sponsorship (M = 4.87 vs. M = 4.67; B = 0.21, β = 0.08, *p* = 0.024).

### Event-sponsor fit

Overall, participants held weaker perceptions of event-sponsor fit for the unhealthy food sponsor brand when compared to both the healthier food sponsor brand (M = 3.61 vs. M = 4.78; t(1131) = − 20.47, *p* < 0.001) and the obesity prevention campaign brand (M = 3.61 vs. M = 4.84; t(1131) = − 21.79, *p* < 0.001). As shown in Table 4, participants exposed to unhealthy food sponsorship perceived a stronger fit between the Commonwealth Games and the unhealthy food sponsor brand than participants exposed to non-food sponsorship (supporting H1c; B = 0.61, β = 0.16; *p* < 0.001). Unexpectedly, participants in the healthier food sponsorship (B = 0.37, β = 0.09; *p* = 0.004) and obesity prevention campaign sponsorship (B = 0.29, β = 0.07; *p* = 0.023) conditions also reported higher levels of event-sponsor fit for the unhealthy food sponsor brand than those in the non-food sponsorship condition.

**Table 4:** Event-sponsor fit perceptions of sponsor brands by sponsorship condition

	Unhealthy food sponsor brand				Healthier food sponsor brand				Obesity prevention campaign sponsor brand			
Sponsorship condition	Predicted mean	B (95% CI)	β	p	Predicted mean	B (95% CI)	β	p	Predicted mean	B (95% CI)	β	p
Non-food branding	3.29	Ref			4.62	Ref			4.70	Ref		
Unhealthy food branding	**3.90**	**0.61 (0.36, 0.86)**	**0.16**	**< 0.001**	4.70	0.08 (−0.15, 0.31)	0.02	0.494	4.72	0.02 (−0.22, 0.25)	0.01	0.872
Healthier food branding	**3.66**	**0.37 (0.12, 0.62)**	**0.09**	**0.004**	**5.10**	**0.48 (0.25, 0.72)**	**0.14**	**< 0.001**	4.77	0.07 (−0.16, 0.31)	0.02	0.539
Obesity prevention campaign branding	**3.58**	**0.29 (0.04, 0.54)**	**0.07**	**0.023**	4.73	0.12 (−0.11, 0.35)	0.04	0.316	**5.19**	**0.49 (0.25, 0.73)**	**0.14**	**< 0.001**

*B* unstandardised regression coefficient, *CI* confidence interval, *β* standardised regression coefficient, *Ref* reference category in linear regression model. Linear regression analyses included product category as a covariate. Boldfaced results are significant at *p* < 0.05

As predicted (H2c), perceptions of fit between the Commonwealth Games and the healthier food sponsor brand were stronger among participants in the healthier food sponsorship condition (B = 0.48, β = 0.14; *p* < 0.001). Similarly, exposure to obesity prevention campaign sponsorship was associated with a higher level of event-sponsor fit for the obesity prevention campaign brand compared to participants exposed to non-food sponsorship (supporting H3b; B = 0.49, β = 0.14; *p* < 0.001).

### Event/brand image similarity

Table 5 shows that participants exposed to unhealthy food sponsorship perceived higher event/brand similarity between the Commonwealth Games and the unhealthy food sponsor brand than participants exposed to non-food sponsorship (supporting H1d; B = 3.07, β = 0.10; *p* = 0.006). Further, as hypothesised (H2d), there was greater concordance between participants’ image perceptions of the Commonwealth Games and healthier food sponsor brand among those who had been exposed to healthier food sponsorship as opposed to non-food sponsorship (B = 2.58, β = 0.09, *p* = 0.010).

**Table 5:** Event/brand image similarity scores^a^ for pairings of Commonwealth Games with sponsor brands^~^ by sponsorship condition

	Commonwealth Games and unhealthy food sponsor brand pairing				Commonwealth Games and healthier food sponsor brand pairing			
Sponsorship condition	Predicted mean	B (95% CI)	β	p	Predicted mean	B (95% CI)	β	p
Non-food branding	37.51	Ref			41.31	Ref		
Unhealthy food branding	**40.58**	**3.07 (0.89, 5.25)**	**0.10**	**0.006**	41.68	0.37 (−1.59, 2.32)	0.01	0.713
Healthier food branding	39.68	2.17 (−0.03, 4.37)	0.07	0.053	**43.89**	**2.58 (0.61, 4.55)**	**0.09**	**0.010**
Obesity prevention campaign branding	38.24	0.74 (−1.46, 2.93)	0.02	0.511	41.69	0.38 (−1.58, 2.34)	0.01	0.704

^a^Sum of the absolute differences in participants’ ratings of the Commonwealth Games and the (a) unhealthy food sponsor brand and (b) healthier food sponsor brand, on 10 adjectives. Scores have been reverse coded such that higher numbers indicate greater image similarity for each event-brand pairing. ^~^Image perceptions of the obesity prevention campaign brands were not assessed

*B* unstandardised regression coefficient, *CI* confidence interval, *β* standardised regression coefficient, *Ref* reference category in linear regression model. Linear regression analyses included product category as a covariate. Boldfaced results are significant at *p* < 0.05

### Brand preferences

**Fig. 3:**
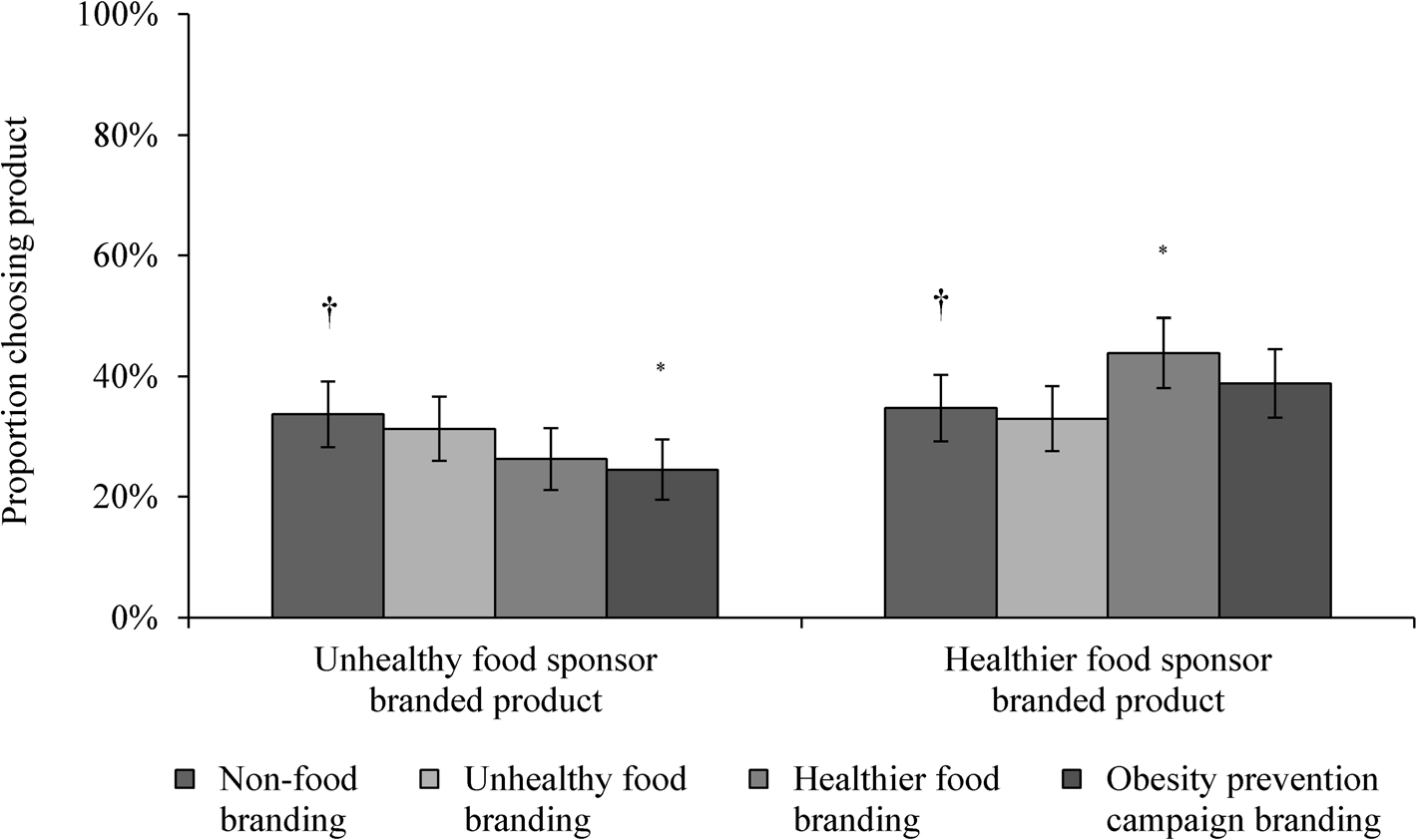
Predicted proportion of young adults who chose sponsor products by sponsorship condition. *Notes*: ^†^ reference category for logistic regression analyses; ^*^ *p* < 0.05; ^**^ *p* < 0.01; ^***^ *p* < 0.001. Logistic regression analyses included product category as a covariate.

As illustrated in Fig. 3, similar proportions of participants in the unhealthy food sponsorship and non-food sponsorship conditions indicated a preference for the unhealthy food sponsor branded product (rejecting H1e; 31.3% vs. 33.7%; OR = 0.89, 95% CI: 0.63–1.27, *p* = 0.531). Participants exposed to healthier food sponsorship were, however, more likely to choose the healthier food sponsor branded product than those exposed to non-food sponsorship (supporting H2e; 43.9% vs. 34.7%; OR = 1.47, 95% CI: 1.05–2.07, *p* = 0.026). Participants exposed to obesity prevention campaign sponsorship showed reduced preference for the unhealthy food sponsor branded product compared to those who saw non-food sponsorship (24.5% vs. 33.7%; OR = 0.63, 95% CI: 0.44–0.92, *p* = 0.016). See Fig. 3.

### Overall preference for unhealthy vs. healthier foods

Across conditions, participants tended to select a higher total number of healthier food products than unhealthy food products in the brand preferences task (M = 1.65 vs. M = 1.35; t(1131) = 5.58, *p* < 0.001). In addressing RQ1b, participants in the obesity prevention campaign sponsorship condition did not show a reduced preference for unhealthy foods compared to those in the non-food sponsorship condition (M = 1.26 vs. M = 1.38; RR = 0.91, 95% CI: 0.79–1.05, *p* = 0.206). There was also no evidence that participants’ overall propensity to choose unhealthy foods was significantly higher following exposure to unhealthy food sponsorship (RQ2; M = 1.43 vs. M = 1.38; RR = 1.03, 95% CI: 0.90–1.19, *p* = 0.627), or significantly lower following exposure to healthier food sponsorship (RQ3; M = 1.31 vs. M = 1.38; RR = 0.95, 95% CI: 0.82–1.10, *p* = 0.478).

## Discussion

The present study provides evidence that young adults were influenced by brief exposure to branded sponsorship of an elite sporting event under simulated conditions, with effects found for both unhealthy and healthier sponsor brands. In line with previous research [14, 15], our findings indicate that sport sponsorship can boost brand awareness, as well as the image and appeal of sponsor brands, ultimately impacting product preferences.

### Brand awareness

Exposure to sport sponsorship for featured brands resulted in significant increases in the proportion of respondents who were aware of these brands across the respective sponsorship conditions, echoing findings of previous sponsorship research [14]. As might be expected given the current food marketing environment where there is a disproportionately high prevalence of unhealthy versus healthier food promotions [2], participants in the control condition showed higher baseline awareness of unhealthy food brands than the healthier food brands and obesity prevention campaign brands. However, the lesser known, healthier brands achieved a larger increase in awareness following the sponsorship intervention, suggesting that sport sponsorship may be an effective way to build public awareness of such brands. The greater boost in awareness resulting from sport sponsorship exposure for the healthier brands may be attributable to these brands being more salient due to less prior exposure, or because they were perceived to be more plausibly associated with the healthier aura of sport. Future research could explore the exact mechanisms underlying such responses.

### Brand attitudes and image

Similar to previous research showing that sport sponsorship can help strengthen brand image and attachment [14, 20, 22, 23], we also found that sport sponsorship exposure promoted more favourable attitudes to featured sponsor brands for both unhealthy and healthier food brands. Notably, healthier food brands were generally perceived more favourably than unhealthy brands, and brief sponsorship exposure to healthier sponsorship further enhanced this difference.

Findings support the contention that sport sponsorship adds a ‘health halo’ to unhealthy food brands. Following exposure to unhealthy food sponsor brands, participants’ perceptions of the image of the sporting event and the brand were more closely aligned, and event-sponsor fit was perceived to be higher. Healthier food sponsorship also promoted greater concordance between perceptions of the event and healthier sponsor brands, and greater perceived event-sponsor fit for healthier food sponsor brands. This suggests that sponsorship could be used to promote the image and appeal of these products. An unexpected finding was that participants in the healthier food sponsorship and obesity prevention campaign sponsorship conditions also reported higher levels of event-sponsor fit for the unhealthy food sponsor brand than those in the non-food sponsorship condition. This could be because participants’ prior history of sponsorship exposure was for predominantly unhealthy brands, such that they are conditioned to such parings being the norm. Notably, participants perceived greater event-sponsor fit for the healthier food brands than the unhealthy food brands across all conditions, possibly because such an association is more plausible to consumers. This bodes well for pairing sport with healthier food sponsor brands, as greater event-sponsor fit is known to predict effective sponsorship [46]. Furthermore, such relationships may be mutually beneficial for the sporting body and the sponsor brand, with ‘spillover’ effects causing transfer of favourable images from one partner to the other [27]. Conversely, there are likely to be reputational risks for sporting bodies of aligning with brands that may carry negative or unhealthy associations.

### Preferences

We found mixed results in relation to sponsorship impacts on product preferences. As expected, healthier food sponsorship increased preference for healthier food sponsor brands, supporting the notion that an increased ‘real world’ sponsorship presence by healthier food brands could promote increased preference for featured brands. However, contrary to expectations, we did not find evidence that unhealthy food sponsorship impacted preference for these foods. We also failed to find impacts of unhealthy food sponsorship exposure on unhealthy food preferences in our previous experimental study assessing impacts of junior sport sponsorship on children [47]. The observed lack of effect of unhealthy food sponsorship on brand preferences could be due to ceiling effects of high prior exposure to advertising for these brands, or that brief sponsorship exposure was insufficient to yield measurable change on this outcome. Given the observed impact of unhealthy food sponsorship on more ‘upstream’ variables such as brand awareness and attitudes, it seems plausible that more cumulative exposure to unhealthy food sponsorship could impact brand preferences over time. However, it is also possible that unhealthy food sponsorship was less persuasive in terms of impact on preferences due to lower perceived event-sponsor fit than for the healthier food sponsor pairings. That the equivalent ‘dose’ of sponsorship for the respective healthier and unhealthy food sponsor conditions differentially impacted preferences suggests that additional processes beyond mere exposure drive sponsorship impacts, as some previous researchers have argued [25]. It was also notable that obesity prevention campaign sponsorship appeared to act as counter-advertising in relation to unhealthy food brands by leading to reduced preference for unhealthy food sponsor brands, which is consistent with the results of our earlier sport sponsorship experiment with children [47].

From the perspective of public health, promoting healthier eating behaviours generally (rather than a preference for particular brands) is paramount. We found that where sponsorship impacted attitudes or preferences, it tended to be brand-specific, rather than impacting overall preference for unhealthy versus healthier foods. Given the large volume of promotions for unhealthy foods to which the public is regularly exposed, this brief sponsorship intervention may have been insufficient to elicit changes in more general food choice behaviours. Indeed, it may have been unrealistic to expect a single exposure to a brief sponsorship message to change overall food preferences, given that these preferences are likely shaped by a lifetime of experience and marketing exposure. To promote healthier eating through sport sponsorship, it may be necessary for events to be sponsored by multiple healthier food brands, and for such exposure to accumulate in volume over an extended period of time.

The failure of obesity prevention campaign sponsorship branding to impact young adults’ food attitudes or overall preference for unhealthy versus healthier foods in this study may likewise be due to participants being unfamiliar with these campaigns and their health messages – LiveLighter only runs in some Australian states, Rethink Sugary Drink does not have a big national profile, while Go for 2&5 is no longer active. Seeing the logos for these campaigns alone (without exposure to supporting campaign messages) was likely insufficient to impact overall preference for unhealthy/healthier food. Further research employing a more cumulative ‘dose’ of sponsorship intervention (e.g. repeated throughout a sports season) and more comprehensive assessment of dietary behaviour post-intervention would improve our understanding of how various sponsorship scenarios impact the overall quality of people’s diets.

### Limitations

There are strengths and limitations to the methods used. Conducting the study online reduced costs and burdens associated with mail-out or phone surveys, enabled us to show videos and access participants in real-time so reactions to sponsorship stimuli could be immediately assessed, and allowed us to reach young adults who can be a challenging population segment to reach using other methods (in Australia, young adults have near universal access to the internet (98% of Australians aged 18–34 years are internet users [48]). However, we were only able to test product preferences in a *simulated* food choice task. Future research could expand on this study by employing methods that allow impacts on *actual* food choices to be assessed. Also, samples recruited from non-probability based online panels have limitations in terms of representativeness. It is possible that severely disadvantaged persons were less likely to participate in our online study. We set quotas to achieve adequate representation of key demographic sub-groups within our sample of young adults and randomly assigned participants to conditions, so baseline characteristics should not have confounded the observed sponsorship effects. While our area-based measure of SEP could not account for variation within postcodes [49], our measure of individual educational attainment indicated our sample had a typical profile to other young Australian adults, with eight out of ten having completed secondary school [50]. Overall our findings align with previous sponsorship research with different populations, suggesting the sponsorship effects we observed are not unique to our participant sample. Employing an experimental design with a large sample of participants enabled us to systematically test how different sponsorship scenarios impacted relative to a control condition. We deliberately employed a sponsorship manipulation that used actual audio-visual promotional material for a high profile, elite sporting event that featured genuine sports stars. This should have facilitated engagement with the sponsorship stimuli and aided processes thought to be critical to achieving sponsorship effects such as image transfer. Using this more intensive, realistic sponsorship simulation, we found clearer evidence of sponsorship impacts than in an earlier sponsorship experiment with children where we only manipulated branded sponsor content featuring static images of sports merchandise with no images of sports stars [47]. Future research could examine whether the effects of various sponsorship scenarios simulated more realistically in the present study occur for other age groups.

### Study implications

The obesity epidemic is a complex public health problem, requiring comprehensive, multi-faceted interventions. Potential regulatory methods for promoting population-level changes in eating behaviour, include taxation and advertising restrictions. To date, most international examples of using regulation to restrict food marketing have focused on direct forms of advertising (e.g. TV product advertisements; on-pack promotions) and on protecting children [51], without constraining the marketing of unhealthy foods through sport sponsorship or considering food marketing impacts on adults. This study documents the impacts of unhealthy food sport sponsorship on young adults and provides evidence on the potential efficacy of various alternative, pro-health sponsorship scenarios. Sport sponsorship promotes awareness of, and favourable attitudes towards, sponsor brands, with brief exposure to healthier food sponsor brands also impacting product preferences. Elite sport sponsorship branding is a potentially modifiable environmental factor that could be reconfigured to promote healthier eating and lifestyle in place of the unhealthy food brands that currently dominate this marketing channel. Restricting sport sponsorship to healthier food brands that meet set nutritional criteria could help promote such foods to young adults in place of unhealthy food brands, whilst retaining sponsorship income for sporting organisations. Our findings complement earlier research [19] and indicate that healthier food brands and obesity prevention campaign brands may have better perceived event-sponsor fit than unhealthy food brands. Public perceptions of the acceptability of such brands as sponsors attest to the feasibility of elite sporting organisations transitioning to healthier sponsor brands.

By focusing on young adults, this study also helps broaden our understanding of how food marketing impacts populations other than children. Vast numbers of young people are exposed to unhealthy product marketing through sport sponsorship. This study suggests that these exposures are likely to be having a real impact on spectators’ awareness, attitudes and preferences for sponsor brands. Findings also highlight the potential utility of using sport sponsorship to promote public health campaigns and healthier food brands to young people.

Given the massive appeal of elite sport in Australian culture [7], there is considerable potential to achieve broad population reach through elite sport sponsorship. In the interests of public health, elite sporting bodies should be encouraged to seek sponsorship from companies wishing to market healthier brands through elite sport. This could be implemented through regulation or voluntarily by sporting organisations. While regulatory bans on elite sport sponsorship by tobacco companies have occurred, bans on sponsorship by unhealthy food companies are unlikely in the short term. There is a need to explore the utility of initiatives that could be implemented more rapidly than regulatory changes. As elite sport competitions and events are dependent on sponsorship to remain viable and profitable, policy action to restrict unhealthy food promotions and branding in sport settings must consider the need for replacement funding sources. Encouraging sponsorship managers to contract these alternative types of brands as sponsors in place of unhealthy food brands could provide a feasible pro-health sponsorship alternative, while maintaining desired profitability. Findings from this research could also inform companies that produce healthier foods of the potential utility of investing in sport sponsorship as a marketing channel for their brands. Healthier sponsorship criteria could be promoted and implemented in sporting organisations to help them build healthier sponsorship arrangements and phase out unhealthy food sponsors. Where an existing sponsor company owns multiple food brands, they could be encouraged to reorient their sponsorship arrangements towards promoting their healthier brands in place of their unhealthy brands (e.g. bottled water brand in place of sugary drink brand). Giving unhealthy food brands less exposure through sport sponsorship should help minimise potential negative impacts on consumers’ diets.

## Conclusions

This experiment provides much needed empirical evidence regarding the efficacy of public health policies aimed at reconfiguring the current unhealthy sport sponsorship environment. We tested the promise of replacing unhealthy food sponsors with either healthier food sponsorship or obesity prevention campaign sponsorship to promote healthier foods to young adults. Findings on how various sponsorship scenarios simulated in this brief intervention impacted brand awareness, image, attitudes and brand preferences suggest that replacing unhealthy food sponsorship with healthier food or non-food brands is a promising intervention.

## Abbreviation

SEP: Socio-economic position
